# Impact of weight loss on survival after chemoradiation for locally advanced head and neck Cancer: secondary results of a randomized phase III trial (SAKK 10/94)

**DOI:** 10.1186/s13014-014-0319-y

**Published:** 2015-01-17

**Authors:** Pirus Ghadjar, Stefanie Hayoz, Frank Zimmermann, Stephan Bodis, David Kaul, Harun Badakhshi, Jacques Bernier, Gabriela Studer, Ludwig Plasswilm, Volker Budach, Daniel M Aebersold, For the Swiss Group for Clinical Cancer Research (SAKK)

**Affiliations:** Department of Radiation Oncology, Charité Universitätsmedizin Berlin, Berlin, Germany; SAKK Coordinating Center, Bern, Switzerland; Department of Radiation Oncology, University Hospital Basel, Basel, Switzerland; Department of Radiation Oncology, Kantonsspital Aarau, Aarau, Switzerland; Department of Radiation Oncology, Clinique de Genolier, Genolier, Switzerland; Department of Radiation Oncology, University Hospital Zürich, Zürich, Switzerland; Department of Radiation Oncology, Kantonsspital St. Gallen, St. Gallen, Switzerland; Department of Radiation Oncology, Inselspital, Bern University Hospital, and University of Bern, Bern, Switzerland

**Keywords:** Weight loss, Chemotherapy, Cisplatin, Head and neck cancer, Hyperfractionated radiation therapy, Survival, Malnutrition

## Abstract

**Background:**

To analyze the impact of weight loss before and during chemoradiation on survival outcomes in patients with locally advanced head and neck cancer.

**Methods:**

From 07/1994-07/2000 a total of 224 patients with squamous cell carcinoma of the head and neck were randomized to either hyperfractionated radiation therapy alone or the same radiation therapy combined with two cycles of concomitant cisplatin. The primary endpoint was time to any treatment failure (TTF); secondary endpoints were locoregional recurrence-free survival (LRRFS), distant metastasis-free survival (DMFS) and overall survival (OS). Patient weight was measured 6 months before treatment, at treatment start and treatment end.

**Results:**

The proportion of patients with >5% weight loss was 32% before, and 51% during treatment, and the proportion of patients with >10% weight loss was 12% before, and 17% during treatment. After a median follow-up of 9.5 years (range, 0.1 – 15.4 years) weight loss before treatment was associated with decreased TTF, LRRFS, DMFS, cancer specific survival and OS in a multivariable analysis. However, weight loss during treatment was not associated with survival outcomes.

**Conclusions:**

Weight loss before and during chemoradiation was commonly observed. Weight loss before but not during treatment was associated with worse survival.

## Background

Weight loss is frequently observed in patients with head and neck squamous cell cancer (HNSCC). Weight loss before chemoradiation has been associated with decreased overall survival by us and others [1-3], however, weight loss during chemoradiation was also associated with poor cancer specific survival, overall survival and disease-free survival in HNSCC [3-5]. Here we report detailed weight loss data before and during chemoradiation and its associations with survival outcomes using long-term follow-up data from our prospective trial testing concomitant cisplatin and hyperfractionated radiation therapy (RT) vs. hyperfractionated RT alone in advanced HNSCC.

## Methods

### Patient selection and treatment

As previously described [1], all eligible patients had invasive HNSCC of the oral cavity, oro- or hypopharynx, or larynx. All patients gave informed consent and a total of 224 patients were randomized between July 1994 and July 2000.

Patients were treated with 1.2 Gy RT twice daily with an interfraction interval of at least 6 hours, 5 days per week, up to a median total dose of 74.4 Gy (range, 72 – 76.8 Gy). In patients of the combined arm cisplatin 20 mg/m^2^ was administered with intravenous hydration on 5 consecutive days during weeks 1 and 5 or 6 of RT, 1.5 hours before the afternoon RT session.

The trial was registered at the National Institutes of Health (www.clinicaltrials.gov; identifier number: NCT00002654) and was approved by the ethics committees of all participating institutions.

### Endpoints and statistics

Patient weight was measured 6 months before treatment (patients were asked to recall their weight 6 months before), at treatment start, before the second cycle and at the end of treatment. Weight loss before treatment was defined as the weight loss from 6 months before treatment start until treatment start. Weight loss during treatment was defined as the maximal weight loss from treatment start to the beginning of the second cycle and the end of treatment. Weight loss was categorized to “no weight loss”, “≤5% weight loss”, “>5-10% weight loss” and “>10% weight loss” [6].

The primary endpoint was time to any treatment failure (TTF). Secondary endpoints included time to local or regional treatment failure (LRRFS), distant metastasis-free survival (DMFS) and overall survival (OS).

All time-to-event end-points were calculated from the date of randomization and were tested for differences using the log-rank test. Treatment failure was defined as either tumor recurrence at any site, salvage surgery, second primary tumor, or death of any cause which ever occurred first. Locoregional failure was defined as local or nodal progression or recurrence or death as a result of tumor. Diagnosis of distant metastasis and death as a result of tumor were considered as events in the time to distant metastasis analysis. Uni- and multivariable Cox regressions were performed to assess prognostic variables. In addition to treatment arm, the following clinical and pathological variables were considered as covariates in the regression models: gender (female vs. male), performance status (WHO grade 0 vs. grade 1–2), primary tumor site (other sites vs. hypopharynx), tumor classification (cT1-2 vs. cT3-4), nodal classification (cN0-1 vs. cN2-3), age (<50 years vs. ≥50 years), acute or late dysphagia (grade 0–2 vs. grade ≥ 3). Categorical endpoints were compared using Fisher’s exact test. As no adjustment for multiple testing was applied all analyses were exploratory and hypothesis generating. Statistical analyses were performed using SAS version 9.2 (SAS Institute Inc., Cary, NC, USA).

## Results

### Incidence and magnitude of weight loss

Patient weight loss at different time points was summarized in Table 1. The proportion of patients with >5% weight loss was 32% before, and 51% during treatment, and the proportion of patients with >10% weight loss was 12% before, and 17% during treatment. In the 57% of patients who did not lose >5% weight before treatment the proportion of patients having >5% and >10% weight loss during treatment was 67% and 44% while in the 32% of patients who had lost >5% weight before treatment 63% and 31% lost >5% and >10% weight, respectively. For 11% of the patients the weight loss before treatment could not be assessed. A total of 6 patients (3%) underwent parenteral nutrition and 72 patients (32%) received a feeding tube.

**Table 1:** **Incidence and magnitude of weight loss**

	**Total (N = 224)**
**Variable**	**n (%)**
Weight loss before treatment start	
No weight loss	78 (34.8%)
<=5% weight loss	49 (21.9%)
>5-10% weight loss	45 (20.1%)
>10% weight loss	27 (12.1%)
Missing	25 (11.2%)
Weight loss during treatment	
No weight loss	34 (15.2%)
<=5% weight loss	73 (32.6%)
>5-10% weight loss	75 (33.5%)
>10% weight loss	39 (17.4%)
Missing	3 (1.3%)

### Clinical predictors of weight loss

Weight loss before treatment was associated with age <50 years (p = 0.006), WHO performance score above 0 (p = 0.004), lower baseline hemoglobin values (p < 0.001), primary tumor site other than hypopharynx (p = 0.04) but not with the baseline total white blood count (p = 0.5). There was a trend between advanced T classification (p = 0.06), N classification (p = 0.07) and male gender (p = 0.09) and increased weight loss before treatment, respectively (Table 2). Weight loss during treatment was associated with treatment arm B (RT alone; p = 0.05) but not with any tested clinical variable including acute dysphagia (Table 3).

**Table 2:** **Clinical predictors for weight loss before treatment**

**Weight loss before treatment**					
	**No weight loss**	**<=5% weight loss**	**>5-10% weight loss**	**>10% weight loss**	p-value*
Sex = Female	15 (46.9%)	4 (12.5%)	11 (34.4%)	2 (6.2%)	0.09
Sex = Male	63 (37.7%)	45 (26.9%)	34 (20.4%)	25 (15.0%)	
WHO performance = 0	51 (44.3%)	33 (28.7%)	23 (20.0%)	8 (7.0%)	0.004
WHO performance > = 1	27 (32.1%)	16 (19.0%)	22 (26.2%)	19 (22.6%)	
Age < 50 years	21 (38.2%)	14 (25.5%)	6 (10.9%)	14 (25.5%)	0.006
Age > = 50 years	57 (39.6%)	35 (24.3%)	39 (27.1%)	13 (9.0%)	
Site = Hypopharynx	21 (42.9%)	10 (20.4%)	16 (32.7%)	2 (4.1%)	0.04
Site = Other	57 (38.0%)	39 (26.0%)	29 (19.3%)	25 (16.7%)	
T classification = cT1-2	21 (52.5%)	9 (22.5%)	9 (22.5%)	1 (2.5%)	0.06
T classification = cT3-4	57 (35.8%)	40 (25.2%)	36 (22.6%)	26 (16.4%)	
N classification = cN0-1	41 (50.0%)	18 (22.0%)	15 (18.3%)	8 (9.8%)	0.07
N classification = cN2-3	37 (31.6%)	31 (26.5%)	30 (25.6%)	19 (16.2%)	
Parenteral nutrition or feeding tube = No	48 (39.0%)	35 (28.5%)	22 (17.9%)	18 (14.6%)	0.2
Parenteral nutrition or feeding tube = Yes	30 (40.5%)	14 (18.9%)	22 (29.7%)	8 (10.8%)	
Hemoglobin > = 14 g/dL	52 (48.1%)	30 (27.8%)	21 (19.4%)	5 (4.6%)	<0.001
Hemoglobin < 14 g/dL	26 (28.6%)	19 (20.9%)	24 (26.4%)	22 (24.2%)	

*Fisher‘s Exact test.

**Table 3:** **Clinical predictors for weight loss during treatment**

**Weight loss during treatment**					
	**No weight loss**	**<=5% weight loss**	**>5-10% weight loss**	**>10% weight loss**	p-value*
Sex = Female	7 (21.2%)	8 (24.2%)	13 (39.4%)	5 (15.2%)	0.5
Sex = Male	27 (14.4%)	65 (34.6%)	62 (33.0%)	34 (18.1%)	
WHO performance = 0	17 (13.7%)	36 (29.0%)	43 (34.7%)	28 (22.6%)	0.1
WHO performance > = 1	17 (17.5%)	37 (38.1%)	32 (33.0%)	11 (11.3%)	
Age < 50 years	13 (21.3%)	21 (34.4%)	18 (29.5%)	9 (14.8%)	0.4
Age > = 50 years	21 (13.1%)	52 (32.5%)	57 (35.6%)	30 (18.8%)	
Site = Hypopharynx	10 (18.5%)	17 (31.5%)	15 (27.8%)	12 (22.2%)	0.5
Site = Other	24 (14.4%)	56 (33.5%)	60 (35.9%)	27 (16.2%)	
T classification = cT1-2	4 (10.0%)	14 (35.0%)	15 (37.5%)	7 (17.5%)	0.8
T classification = cT3-4	30 (16.6%)	59 (32.6%)	60 (33.1%)	32 (17.7%)	
N classification = cN0-1	14 (15.1%)	30 (32.3%)	38 (40.9%)	11 (11.8%)	0.1
N classification = cN2-3	20 (15.6%)	43 (33.6%)	37 (28.9%)	28 (21.9%)	
Acute dysphagia = No	16 (16.7%)	38 (39.6%)	27 (28.1%)	15 (15.6%)	0.2
Acute dysphagia = Yes	18 (14.5%)	35 (28.2%)	47 (37.9%)	24 (19.4%)	
Parenteral nutrition or feeding tube = No	24 (16.7%)	51 (35.4%)	44 (30.6%)	25 (17.4%)	0.4
Parenteral nutrition or feeding tube = Yes	9 (11.8%)	22 (28.9%)	31 (40.8%)	14 (18.4%)	
Arm = A (chemoradiation)	20 (18.0%)	44 (39.6%)	30 (27.0%)	17 (15.3%)	0.05
Arm = B (radiation therapy alone)	14 (12.7%)	29 (26.4%)	45 (40.9%)	22 (20.0%)	

*Fisher’s Exact test.

### Weight loss and survival outcomes

**Figure 1:**
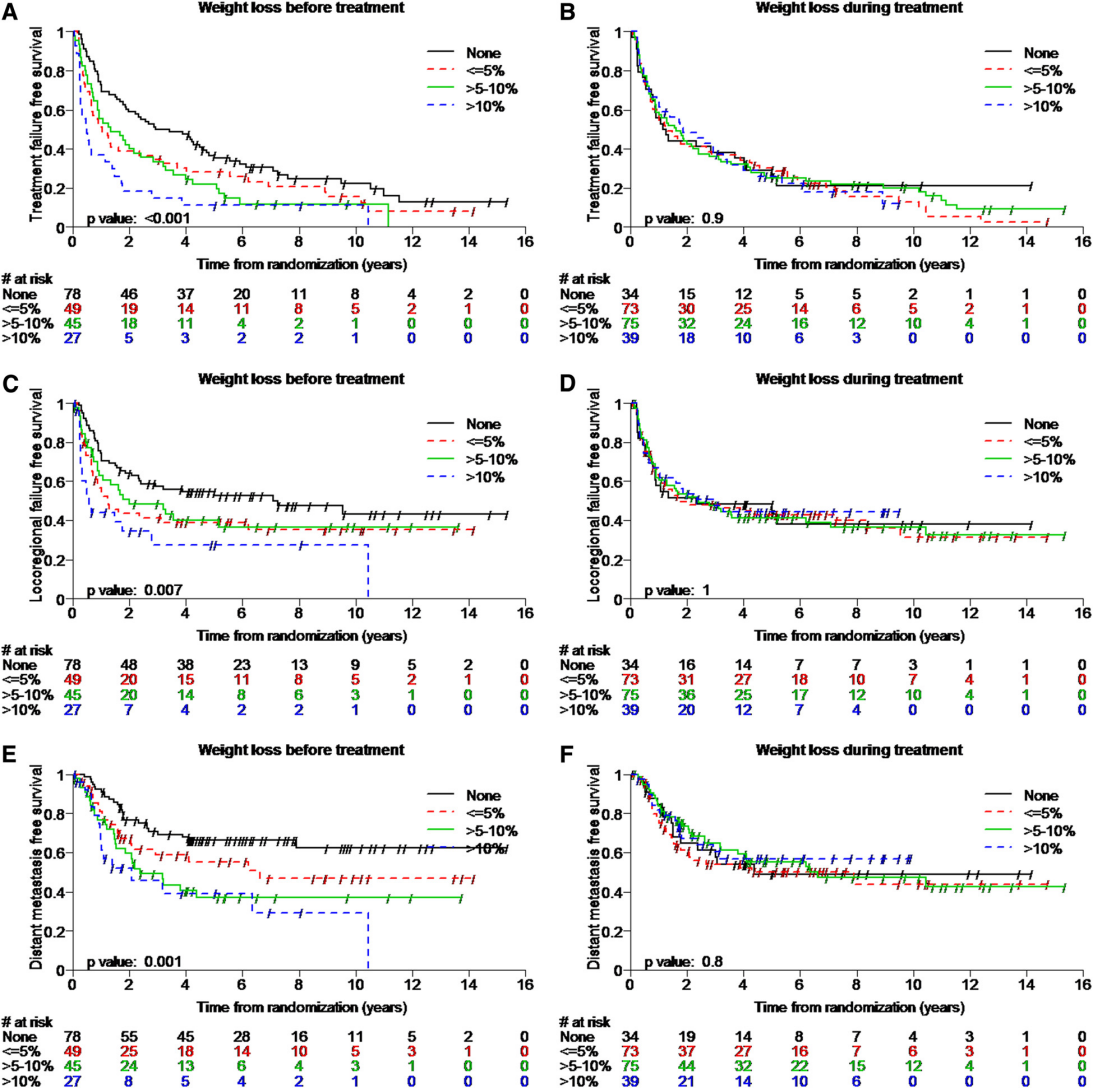
**Kaplan Meier plots showing patients with weight loss before and during treatment and time to any treatment failure (A,B) locoregional failure-free survival (C,D) and distant metastasis-free survival (E,F) over 9.5 years median follow-up stratified according to the magnitude of weight loss.**

**Figure 2:**
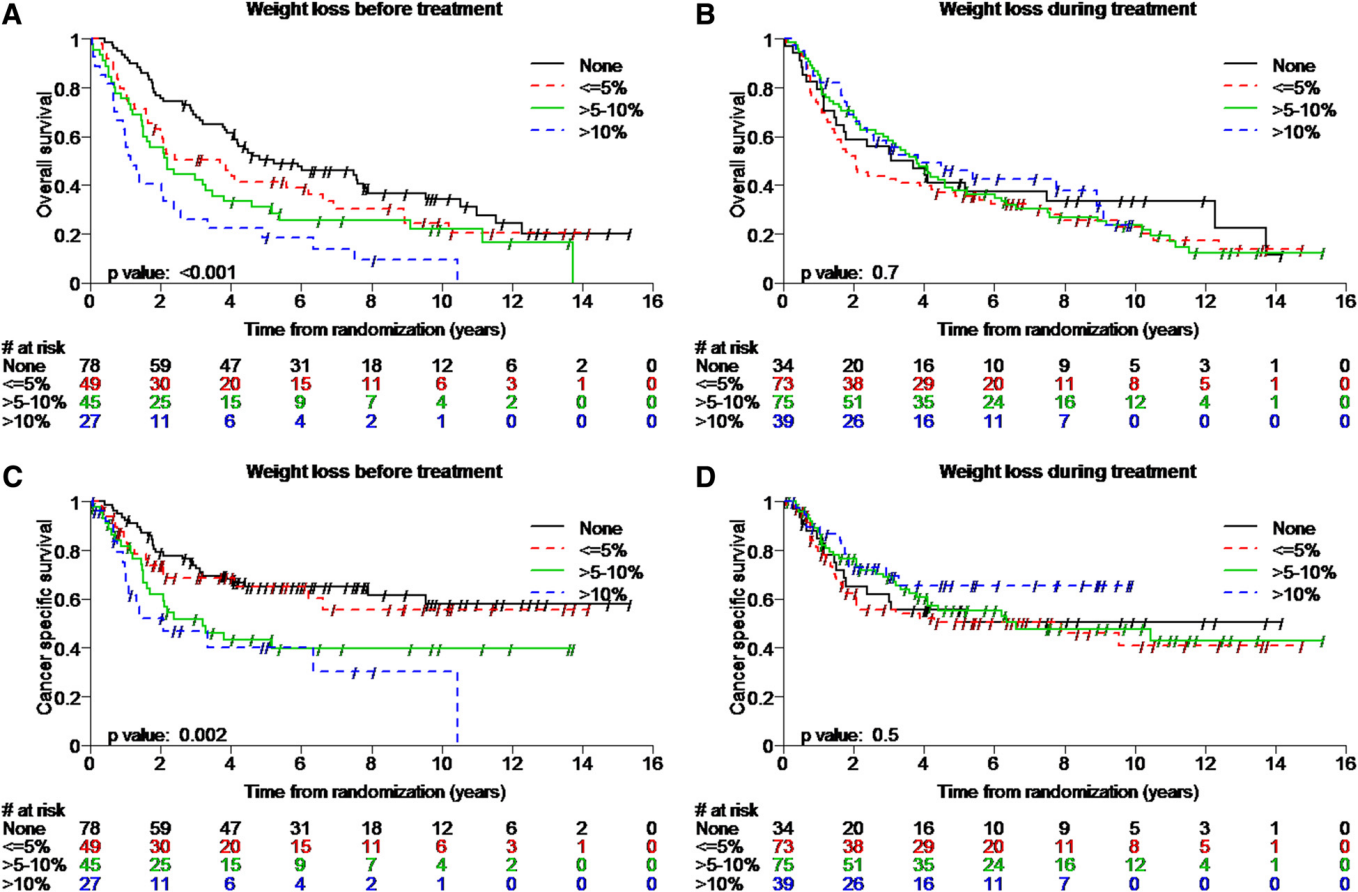
**Kaplan Meier plots showing patients with weight loss before and during treatment and cancer specific survival (A,B) and overall survival (C,D) over 9.5 years median follow-up stratified according to the magnitude of weight loss.**

Median follow-up was 9.5 years (range, 0.1 – 15.4 years). Weight loss before treatment was associated with decreased TTF, LRRFS, DMFS, cancer specific survival (CSS) as well as with decreased OS (Table 4, Figures 1 and 2). After multivariable Cox regression weight loss >10% before treatment remained associated with decreased TTF (hazard ratio [HR], 2.8 [95% confidence interval {CI}, 1.7–4.5; p < 0.0001]), LRRFS (2.5, [95% CI, 1.4–4.5; p = 0.002]), DMFS (HR, 3.1 [95% CI, 1.6–6.1; p < 0.0001]), CSS (HR, 2.8 [95% CI, 1.5–5.4; p = 0.002]), and OS (HR, 3.2 [95% CI, 2.0–5.3; p < 0.0001], (Table 5)).

**Table 4:** **Univariable Cox regression models for the influence of categorized weight loss on survival outcomes**

**Variable**	**Cox regression analysis, hazard ratio (95% confidence interval) (p-value)**				
	**TTF**	**LRRFS**	**DMFS**	**CSS**	**OS**
Weight loss before treatment (<=5% vs. none)	1.5 (1.0-2.2) (0.04)	1.6 (1.0-2.6) (0.05)	1.7 (0.9-2.9) (0.09)	1.2 (0.7-2.2) (0.5)	1.4 (0.9-2.1) (0.1)
Weight loss before treatment (>5-10% vs. none)	1.7 (1.1-2.5) (0.01)	1.4 (0.9-2.3) (0.2)	2.3 (1.3-4.0) (0.003)	2.0 (1.2-3.5) (0.01)	1.7 (1.1-2.7) (0.01)
Weight loss before treatment (>10% vs. none)	2.7 (1.7-4.4) (<0.001)	2.6 (1.5-4.5) (<0.001)	3.1 (1.6-5.8) (<0.001)	2.8 (1.5-5.4) (0.001)	2.9 (1.8-4.8) (<0.001)
Weight loss during treatment (<=5% vs. none)	1.1 (0.7-1.7) (0.7)	1.0 (0.6-1.8) (0.9)	1.1 (0.6-2.1) (0.7)	1.2 (0.6-2.1) (0.6)	1.2 (0.8-2.0) (0.4)
Weight loss during treatment (>5-10% vs. none)	1.0 (0.6-1.6) (1.0)	1.0 (0.6-1.7) (0.9)	0.9 (0.5-1.7) (0.9)	1.0 (0.5-1.8) (0.9)	1.1 (0.7-1.8) (0.7)
Weight loss during treatment (>10% vs. none)	1.1 (0.6-1.8) (0.8)	0.9 (0.5-1.7) (0.8)	0.9 (0.4-1.8) (0.7)	0.7 (0.3-1.5) (0.3)	0.9 (0.5-1.7) (0.8)

p-values for log-rank tests are displayed in the Kaplan-Meier Plots.

**Table 5:** **Multivariable Cox regression models for the influence of categorized weight loss on survival outcomes**

**Variable**	**Cox regression analysis, hazard ratio (95% confidence interval) (p-value)**				
	**TTF**	**LRRFS**	**DMFS**	**CSS**	**OS**
Multivariable analysis*					
Treatment arm: RT only vs. combined	1.3 (1.0-1.8) (0.08)	1.6 (1.1-2.4) (0.02)	1.9 (1.2-3.0) (0.006)	1.9 (1.2-3.0) (0.006)	1.4 (1.0-2.0) (0.05)
Performance status: 1 + 2 vs. 0		1.5 (1.0-2.2) (0.06)	1.6 (1.0-2.4) (0.05)	1.8 (0.9-3.3) (0.08)	
Tumor classification: cT3-4 vs. cT1-2					
Nodal classification: cN2-3 vs. cN0-1	1.3 (0.9-1.8) (0.1)	1.4 (1.0-2.1) (0.08)	1.5 (1.0-2.4) (0.07)	1.5 (1.0-2.4) (0.07)	
Weight loss before treatment (<=5% vs. none)	1.4 (0.9-2.1) (0.12)	1.5 (0.9-2.5) (0.08)	1.6 (0.9-2.9) (0.1)	1.1 (0.6-1.9) (0.9)	1.3 (0.9-2.0) (0.2)
Weight loss before treatment (>5-10% vs. none)	1.5 (1.0-2.3) (0.06)	1.2 (0.7-2.0) (0.5)	1.8 (1.0-3.2) (0.03)	1.6 (0.9-2.9) (0.08)	1.7 (1.1-2.6) (0.02)
Weight loss before treatment (>10% vs. none)	2.8 (1.7-4.5) (<0.001)	2.5 (1.4-4.5) (0.002)	3.1 (1.6-6.1) (<0.001)	2.8 (1.5-5.4) (0.002)	3.2 (2.0-5.3) (<0.001)

*after backward selection with level 0.1.

Weight loss during treatment, however was not associated with any of the tested survival endpoints (Table 4, Figures 1 and 2).

## Discussion

Involuntary weight loss is a major problem in patients with locally advanced HNSCC. The reasons for weight loss include difficulties with oral intake due to tumor-related symptoms such as mechanical obstruction and pain as well as treatment related toxicities. Moreover, weight loss might also cause tumor-associated cachexia, a condition thought to be a multifactorial metabolic disorder. In our trial, the proportion of patients having weight loss >5% was 32% before, and 51% during treatment, and the proportion of patients with >10% weight loss was 12% before, and 17% during treatment. Here we could show that weight loss >10% before treatment is a major predicting factor of decreased survival outcomes after chemoradiation or RT alone. In contrast and interestingly, weight loss during treatment appeared not to be associated with cancer outcome.

Obesity has previously been described to be associated with improved OS in HNSCC and esophageal cancer [7]. For patients with locally advanced HNSCC it has been further reported that pretreatment weight loss ≥10% was associated with decreased OS after multivariable analysis after chemoradiation in a retrospective analysis of a cohort of 92 patients [2]. In this cohort weight loss ≥10% was observed in 49% of patients.

Langius et al. reported on 1340 prospectively collected HNSCC patients, two thirds of those underwent primary RT therapy or chemoradiation, the remaining patients underwent surgery and adjuvant RT therapy. No weight loss before treatment was observed in 70%, ≤5% in 16%, >5-10% in 9% and >10% weight loss in 5% of patients, respectively. During RT weight loss >5% was observed in 57% of patients. Weight loss >10% before treatment remained associated with decreased OS after multivariable analysis and weight loss during RT remained to be associated with decreased disease-specific-survival after multivariable analysis [3].

In a large retrospective cohort of 1562 patients with HNSCC in which two thirds underwent surgery and adjuvant RT and the remaining patients primary chemoradiation Pai et al. could describe that decreased pretreatment body mass index (BMI) was associated with decreased CSS and OS [4]. Overall body weight loss during RT did not influence survival outcomes (39% of patients experienced a body weight loss of ≥5% during RT). In the group with higher pretreatment BMI and less weight loss (<5%) during treatment, however CSS, DMFS and OS was better as compared with those patients having greater weight loss (≥5%) during treatment.

Cho et al. reported on 226 retrospectively analyzed patients with HNSCC of which 74% underwent surgery and 61% either primary or adjuvant RT [5]. Weight loss ≥10% during the year after initial treatment was observed in 42% of patients. T classification and N classification were significantly associated with the occurrence of weight loss ≥10% in this series. After multivariable analyses, weight loss ≥10% was a predictor of decreased disease-free survival.

The proportion of patients observed with significant weight loss in our trial appears to be within the range reported in the literature and we could gather further indications for the importance of pretreatment weight loss as a major prognostic factor. Interestingly, however, we could find no indications that weight loss during treatment has any prognostic role. This finding might question the benefit of a rigorous strategy against weight loss during treatment. Taking into account both the significant morbidity and costs associated with both, feeding tubes and/or parenteral nutrition, more evidence is warranted to define the optimal nutritional support during chemoradiation of advanced HNSCC and its impact on treatment outcome.

We believe that pretreatment weight loss is most probably caused by cancer-associated cachexia and that it is associated with advanced disease factors and decreased performance status. It might be that an impaired immune response caused by significant pre-treatment weight loss further contributes to the decreased survival outcomes. Interestingly pretreatment weight loss was significantly associated with lower baseline hemoglobin values, a factor which was associated with decreased survival outcomes in a previous analysis of this dataset [1], however, baseline hemoglobin did not remain significant in the multivariable analysis for the different survival endpoints analyzed. One limitation of our study is that smoking habits were not collected as there is an association between weight, weight loss and smoking habits and smoking status might be a confounding factor for mortality. Another limitation of this work is the fact that the pre-treatment weight was asked by recall, which could potentially be associated with some impreciseness.

In a secondary analysis of the Radiation Therapy Oncology Group (RTOG) 90–03 trial which prospectively collected data on nutritional support delivered before treatment, during treatment, and after treatment data of the 1073 evaluable patients were analyzed against outcome. Interestingly, patients receiving pre-treatment nutritional support experienced significantly less weight loss by the end of treatment and less grade 3 to 4 mucositis than patients not receiving pre-treatment nutritional support. Yet patients receiving pre-treatment nutritional support had a poorer 5-year actuarial locoregional control rate and a poorer 5-year overall survival rate [8]. This finding indicates that the mechanism behind the negative impact of pretreatment weight loss is more complex than just mechanical obstruction and pain caused by the primary tumor. It rather points towards biological and metabolic factors associated with the aggressiveness of the primary tumor and/or the tumor load. It is therefore questionable whether the prognosis of these patients can simply be improved by correction of the nutritional status.

Our study confirms within the frame of a prospective randomized trial that patients with significant pretreatment weight loss are at higher risk for treatment failure. So far, no specific intervention has been established to counteract this adverse condition in order to improve patients’ survival. We propose to stick to a tight follow up schedule for these high-risk patients. Moreover, as another consequence of our results, we suggest that future prospective studies in head and neck cancer might benefit from stratification according to pretreatment weight loss.

## Abbreviations

SAKK: Swiss Group for Clinical Cancer Research
TTF: Time to any treatment failure
LRRFS: Locoregional recurrence-free survival
DMFS: Distant metastasis-free survival
OS: Overall survival
HNSCC: Head and neck squamous cell carcinoma
RT: Radiation therapy
HR: Hazard ratio
CI: Confidence interval
BMI: Body mass index
RTOG: Radiation Therapy Oncology Group
